# Invasive treatment of asymptomatic extracranial carotid stenosis. A conceptual approach

**DOI:** 10.1590/1677-5449.170201

**Published:** 2018

**Authors:** Ricardo Cesar Rocha Moreira

**Affiliations:** 1 Hospital Nossa Senhora das Graças, Serviço de Cirurgia Vascular, Curitiba, PR, Brasil.

 In the second decade of the XXI century, a vast body of knowledge (>4,800 articles listed in Medline) has been accumulated on all aspects of extracranial carotid disease. Out of this wealth of information, scientific Societies all over the world have proposed guidelines for the management of cerebrovascular disease. 1 ^-^ 4

 Current guidelines have reached nearly a consensus on the role of invasive treatment in symptomatic patients with extracranial carotid stenosis. 5 However, in asymptomatic patients, there is much uncertainty as to whether invasive treatments - carotid andarterectomy (CEA) and carotid angioplasty with stenting (CAS) - reduce stroke risk. The benefit of adding an invasive treatment to Best Medical Therapy (BMT) is currently the most controversial issue in the management of patients with asymptomatic extracranial carotid stenosis. 6

 The author proposes an alternate approach to the issue of which patients with asymptomatic carotid disease can benefit from invasive treatment. The knowledge accumulated in over a century of studies can be summarized in a few concepts, from which a framework can be formed. 7 The conceptual framework can be used as a provisional tool to make clinical decisions, when guidelines are not clear or applicable to an individual patient. 

 The first concept can be stated as follows: “the vast majority of individuals with extracranial carotid lesions are asymptomatic and will remain asymptomatic for life.” This concept is based on over 50 long-term prospective studies that followed patients with carotid plaques on medical treatment. 5 ^,^ 8 Those studies have shown that patients with asymptomatic carotid atherosclerosis have a much higher risk of ischemic cardiac events and long-term mortality, compared with the population at large. 8 This concept leads to two corollaries: the presence of an asymptomatic carotid plaque is a strong marker of systemic atherosclerosis; and all patients in whom a carotid plaque is detected should be placed on BTM and followed closely for progression of their atherosclerotic disease. 3 ^,^ 4 ^,^ 8

 The second concept refers to risk: “Patients with asymptomatic carotid atherosclerosis on BMT have a low risk of developing an ischemic cerebral vascular accident (CVA).” A recent meta-analysis of 49 long-term studies showed that the overall risk of ischemic brain events in patients on medical management is below 1% per year of follow-up. 9 Nevertheless, a minority of patients with asymptomatic carotid lesions will develop brain ischemia. The quest for physicians involved in care of patients with asymptomatic carotid atherosclerosis is finding which patients are at higher-than-average risk of developing brain ischemic events. 4 ^-^ 6

 The third concept refers to benefit: “The benefit of invasive treatment of asymptomatic extracranial carotid atherosclerosis is limited to a subgroup of patients with high-risk lesions.” Namely, vulnerable carotid plaques. The characteristics that define a vulnerable plaque include: a large lipid-laden core, a thin fibrous cap, inflammation in and/or around the plaque, vasavasorum neovascularization, and intraplaque hemorrhage. 10 Vulnerable plaques are particularly prone to develop plaque events. The most common events: plaque ulceration, intraplaque hemorrhage and plaque rupture result in extrusion of atheromatous contents into the arterial lumen, causing embolization to the distal arterial bed. Distal embolization from events within a carotid plaque is the main mechanism of brain ischemia, and can present clinically as transient ischemic attack (TIA) and ischemic cerebral vascular accident (CVA). 4 ^,^ 10 The other mechanism of brain ischemia related to the plaque is internal carotid artery occlusion, that can be caused by an plaque event such as hemorrhage or by progression of a plaque to high-degree or subocclusive stenosis, with subsequent thrombosis. 10

 A number of studies have been conducted in asymptomatic carotid patients on BMT in order to identify factors that increase the risk of a cerebral ischemic event. 4 ^,^ 11 In those studies, the following clinical or imaging factors have been found to be associated with statistically significant increased risk of late stroke in asymptomatic patients with a 60-99% extracranial carotid stenosis:

- Silent brain infarction on CT scan;
- Progression of degree of stenosis on serial Doppler ultrasonography exams;
- Plaque area in computerized plaque analysis: the larger the plaque, the larger the risk;
- Size of juxta-luminal hypoechoic (or echolucent) area within the carotid plaque;
- Intraplaque hemorrhage on MR imaging;
- Impaired CVR - cerebral vascular reserve - on transcranial ecodoppler;
- Predominantly echolucent plaque on Doppler ultrasonography;
- Spontaneous embolization on transcranial ecodoppler;
- Spontaneous embolization on transcranial ecodoppler, plus echolucent plaque;
- Contralateral carotid occlusion or contralateral clinical cerebral ischemia (TIA or CVA).

Over the next 5 to 10 years, the ongoing prospective studies will provide hard data on the significance of those clinical and imaging risk factors and will probably change current recommendations on the invasive treatment of asymptomatic extracranial carotid stenosis. 12 While this new information is not available, the question “Which patients can benefit of invasive treatment for asymptomatic extracranial carotid stenosis ?” can be answered using the conceptual framework presented above:

1. A carotid atherosclerotic plaque is a strong marker of systemic atherosclerosis. All patients with a significant plaque should be on BMT and followed closely for cardiovascular ischemic events;
2. Invasive treatments - CEA or CAS - should be offered only to asymptomatic patients on BMT whose plaques present with or progress to high-degree/subocclusive stenosis; and to patients in whom clinical or imaging evaluation suggests a vulnerable plaque with high-risk of triggering cerebral ischemia.
