# Supplementary material for: Invasive treatment of asymptomatic extracranial carotid stenosis. A conceptual approach
Source: J Vasc Bras. 2018 Apr-Jun;17(2):101–3. doi: 10.1590/1677-5449.170201 (PMC6205706; doi:10.1590/1677-5449.170201)
Supplement: Translation

# Translation: Tratamento invasivo da estenose assintomática da carótida extracraniana. Uma abordagem conceitual

**Authors:** Ricardo Cesar Rocha Moreira

**Affiliations:** 1 Hospital Nossa Senhora das Graças, Serviço de Cirurgia Vascular, Curitiba, PR, Brasil.

 Nesta segunda década do século XXI, um vasto corpo de conhecimento (> 4,800 artigos listados no MEDLINE) já foi acumulado em todos os aspectos da doença da carótida extracraniana. A partir desta riqueza de informação, Sociedades científicas de todo o mundo vêm propondo diretrizes para o manejo da doença cerebrovascular 1 ^-^ 4 . 

 As diretrizes mais recentes são quase consensuais quanto ao manejo dos pacientes sintomáticos com aterosclerose da carótida extracraniana 5 . Contudo, em pacientes assintomáticos, há muita incerteza se os tratamentos invasivos - endarterectomia da carótida (EAC) e angioplastia da carótida com stent (ACS) - reduzem o risco de isquemia cerebral. O benefício de se indicar um tratamento invasivo além do Tratamento Clínico Otimizado (TCO) é atualmente a questão mais controvertida no manejo de pacientes com estenose assintomática da carótida extracraniana 6 . 

 O autor propõe uma abordagem alternativa para responder à questão de quais são os pacientes com doença carotídea assintomática que podem se beneficiar dos tratamentos invasivos. O conhecimento acumulado ao longo de mais de um século pode ser resumido em uns poucos conceitos, com os quais se pode formar uma estrutura 7 . A estrutura conceitual pode então ser usada como uma ferramenta provisória para a tomada de decisões clínicas, quando diretrizes não são claras ou aplicáveis em um caso em particular. 

 O primeiro conceito pode ser enunciado da seguinte forma: “a vasta maioria dos indivíduos com estenoses da carótida extracraniana são assintomáticos e vão permanecer assintomáticos por toda a vida”. Este conceito é baseado em mais de 50 estudos prospectivos de longo prazo, que seguiram pacientes com placas significativas de carótida sob tratamento clínico 5 ^,^ 8 . Os estudos mostraram que pacientes com estenoses assintomáticas das carótidas tinham um risco elevado de eventos cardíacos isquêmicos e mortalidade alta a longo prazo, comparados com a população em geral 8 . Este conceito leva a dois colorários: a presença de uma placa assintomática de carótida é um forte marcador de aterosclerose sistêmica; e todos os pacientes em quem uma placa de carótida é detectada devem ser submetidos a TCO e seguidos de perto para progressão da sua doença aterosclerótica 3 ^,^ 4 ^,^ 8 . 

 O segundo conceito é referente ao risco: “Pacientes com aterosclerose carotídea assintomática sob TCO têm um risco muito baixo de desenvolver acidente vascular cerebral (AVC) isquêmico”. Uma meta-análise recente de 49 estudos de longo prazo mostra que, no geral, o risco de eventos isquêmicos cerebrais dos pacientes sob tratamento clínico é menos de 1% por ano de seguimento 9 . Mesmo assim, uma minoria dos pacientes com lesões assintomáticas da carótida vão desenvolver isquemia cerebral. A missão dos médicos envolvidos em cuidar de pacientes com estenose assintomática da carótida é descobrir quais pacientes tem risco mais elevado de desenvolver eventos isquêmicos cerebrais 4 ^-^ 6 . 

 O terceiro conceito se refere a benefício: “O benefício do tratamento invasivo da aterosclerose assintomática de carótida extracraniana se limita a um subgrupo de pacientes com lesões de alto risco”. Especificamente, placas de carótida vulneráveis. As características que definem uma placa vulnerável incluem: um núcleo grande rico em lipídios: uma capa fibrose tênue; inflamação dentro ou ao redor da placa; neovascularização nos vasavasorum; e hemorragia intraplaca 10 . Placas vulneráveis são particularmente propensas a desenvolver eventos intraplaca. Os eventos mais comuns: ulceração da placa, hemorragia intraplaca e ruptura da placa resultam em extrusão do conteúdo ateromatoso na luz arterial, causando embolização para o leito arterial distal. Embolização distal a partir de eventos intraplaca é o principal mecanismo de isquemia cerebral, e pode se apresentar clinicamente como com ataque isquêmico transitório (AIT) ou com acidente vascular cerebral isquêmico (AVC) 4 ^,^ 10 . O outro mecanismo de isquemia cerebral relacionado ao evento intraplaca é oclusão da carótida interna, que pode ser causada por um evento intraplaca, como uma hemorragia, ou por progressão da placa para estenose de alto grau/suboclusiva, com trombose subsequente 10 . 

 Uma série de estudos foram conduzidos em pacientes assintomáticos sob TCO para tentar identificar fatores que aumentam o risco de um evento isquêmico cerebral 4 ^,^ 11 . Nestes estudos, os seguintes fatores clínicos e de imagem foram associados com aumento estatisticamente significativo do risco de isquemia tardia em pacientes assintomáticos com estenose da carótida de 60-99%:

- Infarto cerebral silencioso à TAC - tomografia computadorizada;
- Progressão do grau de estenose em exames seriados de ecodoppler;
- Área da placa em análise computadorizada (quanto maior a placa, maior o risco);
- Tamanho da área hipoecóica (ou ecolucente) justaluminal dentro da placa;
- Hemorragia intraplaca na imagem por Ressonância Magnética;
- Reserva vascular cerebral reduzida no ecodoppler transcraniano;
- Placa predominantemente ecolucente no exame de ecodoppler;
- Embolização espontânea no Doppler transcraniano;
- Embolização espontânea no Doppler transcraniano, mais placa ecolucente;
- Oclusão da carótida contralateral ou isquemia cerebral sintomática contralateral.

Nos próximos 5 a 10 anos, os estudos prospectivos em andamento vão gerar dados sólidos quanto ao significado destes fatores clínicos e de imagem, que provavelmente vão mudar as recomendações atuais para o tratamento invasivo da estenose assintomática da carótida extracraniana 12 . Enquanto estas informações novas não estiverem disponíveis, a questão “Quais pacientes podem se beneficiar do tratamento invasivo da estenose assintomática da carótida extracraniana?” pode ser respondida com o uso da estrutura conceitual descrita acima:

1. A placa de ateroma da carótida é um forte marcador de aterosclerose sistêmica. Todos os pacientes com uma placa significativa devem ser colocados em TCO e seguidos cuidadosamente, pelo alto risco de eventos isquêmicos cardiovasculares;
2. Tratamentos invasivos - EAC ou ACS - devem ser indicados apenas em pacientes assintomáticos que se apresentam com ou progridem para estenoses de alto grau/suboclusivas; e em pacientes nos quais a avaliação clínica e de imagem sugerem uma placa vulnerável, com alto risco de provocar isquemia cerebral.
